# Local effect of simvastatin combined with different osteoconductive biomaterials and collagen sponge on new bone formation in critical defects in rat calvaria ^1^

**DOI:** 10.1590/s0102-865020200010000002

**Published:** 2020-03-20

**Authors:** Dircilei Nascimento de Sousa, Virgílio Moreira Roriz, Guilherme José Pimentel Lopes de Oliveira, Wagner Rodrigues Duarte, Leonardo Nogueira de Miranda Pereira Pinto, Leonora Maciel de Souza Vianna, Fabiana Pirani Carneiro, Vania Maria Moraes Ferreira

**Affiliations:** I PhD, Postgraduate Program in Medical Sciences , School of Medicine , Universidade de Brasília (UnB), Brazil . Design, intellectual and scientific content of the study.; II Associate Professor, Faculty of Dentistry , Universidade Federal de Goiás (UFG), Goiania - GO , Brazil . Manuscript writing, critical revision.; III Assistant Professor, Faculty of Dentistry , Universidade Federal de Uberlândia (UFU), Uberlandia - MG , Brazil . Statistical analysis.; IV Associate Professor, Faculty of Dentistry , UnB , Brasilia - DF , Brazil . Design, intellectual and scientific content of the study.; V Graduate student, Faculty of Dentistry , UnB , Brasilia - DF , Brazil . Surgical procedures.; VI Associate Professor, School of Medicine , UnB , Brasilia - DF , Brazil . Histological and histomorphometrical examinations.; VII Associate Professor, Postgraduate Program in Medical Sciences , School of Medicine , UnB , Brasilia - DF , Brazil . Histological and histomorphometrical examinations.; VIII Associate Professor, Postgraduate Program in Medical Sciences , School of Medicine , UnB , Brasilia - DF , Brazil ,. Design, intellectual and scientific content of the study.

**Keywords:** Biocompatible Materials, Bone Regeneration, Simvastatin, Rats

## Abstract

**Purpose:**

To evaluate the local effect of simvastatin (SVT) combined with deproteinized bovine bone (DBB) with hydroxyapatite/β-tricalcium phosphate biphasic ceramics (HA/TCP) and with collagen sponge (CS) on bone repair in critical size defects (CSDs) in rat calvaria.

**Methods:**

Forty-two 5-mm diameter CSDs were made bilaterally in the calvaria of 18 rats. The animals were allocated according to the type of biomaterial and associations used to fill the CSD. After 8 weeks, the animals were euthanized, and their calvaria were evaluated for repaired tissue composition using histologic and histometric analyses.

**Results:**

In the histometric analysis, the use of SVT showed to increase bone formation in the CSDs when combined with all the bone substitutes tested in this study (p<0.05). Greater bone formation was observed in the groups with SVT compared to the groups without SVT.

**Conclusions:**

The use of SVT without the need for a vehicle and combined with a commercially available biomaterial may be a cheaper way to potentiate the formation of bone tissue without the need to produce new biomaterials. Therefore, SVT combined with DBB induced significantly greater new bone formation than did the other treatments.

## Introduction

The identification of bone substitutes that can be used in grafting procedures and have a different origin than autogenous bone grafts is extremely important due to the high prevalence of bone defects that require grafting procedures and the inability to use autogenous bone grafts in all cases. Although this type of graft has some of the best biological properties for bone formation ^1^ , its use is limited by the morbidity caused to the patient, its limited availability and its resorption rates ^2 , 3^ .

Accordingly, osteoconductive biomaterials, such as deproteinized bovine bone (DBB) and hydroxyapatite/β-tricalcium phosphate biphasic ceramics (HA/TCP), have been indicated as alternative biomaterials to the use of autogenous bone grafts in bone grafting procedures ^4 , 5^ . These biomaterials have shown to be effective in increasing bone availability associated with maxillary sinus floor augmentation ^4^ , in alveolar ridge preservation following tooth extraction ^6^ and in lateral ridge augmentation ^7^ . However, in spite of these benefits, the use of strictly osteoconductive biomaterials has shown to reduce the formation of bone tissue in comparison to areas grafted with autogenous bone ^8^ , to reduce bone formation in non-critical size defects compared to those that were filled with clots ^9^ and to have worse implant osseointegration compared to implants placed in native bone ^10^ .

Because of this, some combinations have been suggested to improve the biological properties of osteoconductive biomaterials, such as combining them with autogenous bone graft ^11^ and with growth factors, such as rhBMP2 ^12^ . However, these combinations still present limitations in terms of patient morbidity and a questionable cost-benefit ratio ^3 , 12^ . In addition, growth factors, such as rhBMP2, have been used in conjunction with collagen sponge (CS), which appears to be a more stable scaffold than osteoconductive biomaterials ^13^ despite leaving less space for bone formation during the healing of bone defects ^14^ . Thus, it is important to find alternative combinations to potentiate the formation of bone tissue in areas grafted with osteoconductive bone substitutes.

Simvastatin (SVT), for example, is a drug traditionally used in serum cholesterol control because it inhibits the enzyme 3-hydroxy-3-methylglutaryl coenzyme A reductase (HMG-CoA) ^15 , 16^ . However, this drug has shown to have pleiotropic effects that are related to its anti-inflammatory and connective tissue-proliferating properties ^17 , 18^ . These effects have shown to benefit bone tissue formation both by inhibiting osteoclastogenesis through blocking the mavelonate pathway ^19^ and by stimulating osteoblastic activity through the increase in the expression of bone morphogenic protein (BMP2) ^17^ , vascular endothelial growth factor (VEGF) ^20^ and alkaline phosphatase ^17 , 21^ . These properties make SVT a good alternative as an osteoinductive agent that can improve the biological properties of osteoconductive biomaterials ^18 - 20^ .

The studies that combined the use of SVT with bone substitute biomaterials show contradictory results. Preclinical studies have shown that SVT increased bone formation in non-critical size defects in rabbit femur grafted with DBB and calcium sulphate ^18^ , increased bone formation when combined with a gelatin/nanohydroxyapatite scaffold in rabbit radio defects ^20^ and increased bone formation in critical size defects of rat calvaria when combined with a calcium sulphate scaffold, with SVT being slowly released by polyglycolic acid polymer microspheres ^22^ . However, other studies have shown that the combination with SVT did not promote increased bone formation. A recent clinical study that evaluated the application of a PLGA/HA/β-TCP with SVT scaffolds in maxillary third molar post-extraction sockets demonstrated a high rejection rate ^23^ . Moreover, a preclinical study showed that the use of SVT combined with β-TCP in critical size defects in rat calvaria at concentrations of 0.25 and 0.5 mg did not improve bone tissue formation ^24^ . Thus, it is clear that parameters, such as the type of scaffold or vehicle to be used in conjunction with SVT, as well as adequate dosages of this drug, still need to be determined ^23 , 24^ .

Therefore, the objective of this study was to evaluate the combination of 0.1 mg SVT with different osteoconductive biomaterials (DBB, HA/TCP and CS) in terms of new bone formation in rat calvaria critical size defects (CSDs).

## Methods

The study protocol was approved by the Committee for the Use of Animals in Research, University of Brasilia, Brasilia, DF, Brazil (protocol doc 7838/2015). Twenty-one female Wistar rats (eight weeks of age, average weight of 300 g) were used in this study. The animals were housed in groups of five per cage in standard conditions with food and water *ad libitum* at room temperature with a 12-hour light/dark cycle (06:00 to 18:00 h). Female rats were used in this experiment because previous work from our research group showed very good response stability in the results obtained, although hormonal interferences were not considered ^25^ . The ARRIVE protocol guidelines was followed in this study.

The animals were randomly allocated into six groups. The animals were allocated according to the type of biomaterial and associations used to fill the CSD. A total of 42 defects were created in 18 rats and were divided into 6 groups that included 6 defects in each group as follows: DBB group - CSD filled with DBB (Bio Oss ^®^ Institute Geistlich, Zurique, Switzerland); HA/TCP group - CSD filled with HA/TCP (BoneCeramic ^®^ Institute Straumann AG, Basel, Switzerland); CS group - CSD filled with CS (CollaTape ^®^ Zimmer, Cornellà de Llobregat, Barcelona, Spain); DBB + S group - CSD filled with DBB associated with simvastatin (0.1 mg); HA/TCP + S group – CSD filled with HA/TCP associated with simvastatin (0.1 mg); and CS + S group – CSD filled with CS associated with simvastatin (0.1 mg). One side of the CSD was filled with the pure biomaterial while the other one was filled with the biomaterial combined with simvastatin.

### *Preparation of simvastatin solution*

The SVT solution was prepared and applied to the bone defects as previously described ^26^ . Briefly, a solution containing 0.1 mg of SVT diluted in 15 µL of ethanol was applied to each CSD. The CSD filled with DBB and HA/TCP received 14 mg of the scaffold soaked in 15 µL of the SVT solution. The CS that filled the CSD was soaked in 15 µL of the SVT solution.

### *Surgical procedures*

The animals were anesthetized with a combination of ketamine (80 mg/kg) and xylazine (10 mg/kg) by intramuscular injection. An antiseptic (povidone-iodine) was applied to the surgical sites, a skin incision was performed, and a flap was raised exposing the calvarial bone. Two critical-sized bone defects 5 mm in diameter just lateral to the sagittal plane were carefully prepared with a trephine bur under irrigation with saline solution and slight pressure to avoid damage to the dura mater. After filling the CSDs, the flaps were then sutured using 5-0 nylon suture. Aspirin (150 mg/kg) was given orally to the rats every 6 hours on the first day after surgery. The animals were observed daily for signs of inflammation. The animals were euthanized 8 weeks after the surgical procedure.

### *Histological preparation and histological and histomorphometrical analyses*

After euthanasia, the calvarial bones were dissected, the soft tissues were carefully removed, and the specimens were then fixed in neutral 10% formalin for 24 hours. The specimens were then washed in water for 24 hours and decalcified with a solution of 50% formic acid and 20% sodium citrate for 30 days. The calvarial bones were divided in half longitudinally, and each half containing one treated defect was separately embedded in paraffin according to standard protocols. The embedded specimens were sectioned into 5 µm serial slices with a microtome. All sections were stained with haematoxylin and eosin for subsequent microscopic and histomorphometrical analyses.

Histological analysis was carried out with a light microscope under x20 and x200 magnification, and the morphology of the newly formed tissue in the bone defect area was examined. Tissue sections were screened under a light microscope, and the most central histological sections of each surgical defect were selected for the analyses. The histomorphometrical analysis was carried out using ImageScope ^®^ software, and the area of the newly formed bone and the remaining biomaterial were calculated. Briefly, the total area was delineated on the captured digital images of the entire surgical defects as follows: two vertical lines were drawn on each side of the defect limited by the original cortical calvarial bone. These two vertical lines on each side were connected by two horizontal lines with one on top and another at the bottom, forming a rectangle containing the entire newly formed tissue. The area of this rectangle was considered to be the total area to be analysed. Then, the newly formed bone and the remaining biomaterial particles were selected, and the area was calculated as a percentage of the total area. This analysis was performed by a single blinded evaluator.

### *Statistical analysis*

GraphPad Prism 6 software (San Diego, CA, USA) was used to perform the statistical analysis. The histometric data had a normal distribution confirmed using the Shapiro-Wilk Test. For the inferential analysis, the histometric data were evaluated using a parametric test and one-way ANOVA complemented by the Tukey test to evaluate the different scaffold types (DBB vs. HA/TCP *vs* . CS). A paired t-test was used to compare the scaffolds alone or combined with SVT. All the statistical tests were applied at a significance level of 95%.

## Results

**Figure 1:**
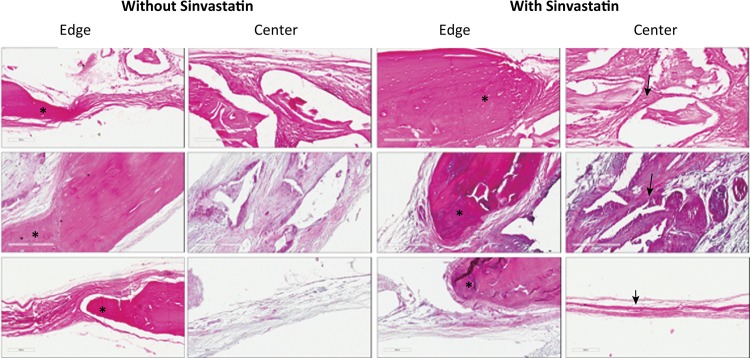
Representative images at the edges and at the center of the defects (HE-x200). The upper panels represent DBB, the middle panels represent HA/TCP, and the lower panels represent CS. At the edges of the defects, it is possible to observe a more robust bone formation at the groups where the SVT was associated with the materials ( * ); At the center of the defects, it was observed a higher organization of the connective matrix associated with a more intense inflammatory infiltrate at the groups where the SVT was associated with the materials ( *black arrows* ).

In accordance with Figure 1 , it is possible to observe the histological description of the obtained results. CSDs grafted with DBB showed greater bone formation at the margins of the defects, where it was possible to observe the presence of mature lamellar bone with flattened osteocytes. Bone was also observed to be associated with biomaterial particles and had a mature appearance. In addition, the presence of well-organized connective tissue with abundant blood vessels and the presence of a mild inflammatory infiltrate was observed. The addition of SVT produced similar histological findings with the exception of the presence of a moderate inflammatory infiltrate.

In HA/TCP group, the formation of bone tissue occurred on the margins of the CSDs and had a mature appearance with the presence of bone lamellae and flattened osteocytes. The presence of immature bone was also observed showing structural disorganization of the matrix and the presence of rounded osteocytes. The connective tissue was organized with a good amount of blood vessels. CSDs grafted with HA/TCP alone showed mild inflammatory infiltrate, whereas a moderate presence of inflammatory cells was observed in the group in which combined HA/TCP and SVT was used to fill the CSD.

The defects filled with CS alone showed abundant inflammatory infiltrate, well-organized connective tissue and bone formation on the margins of the defect, and the bone was at an advanced stage of maturation. When CS was combined with SVT, some areas of bone formation were observed. However, much of the newly formed bone was located in the margin of the defect and was mature. Moreover, well-organized connective tissue was observed with the presence of blood vessels associated with a few inflammatory cells.

**Figure 2:**
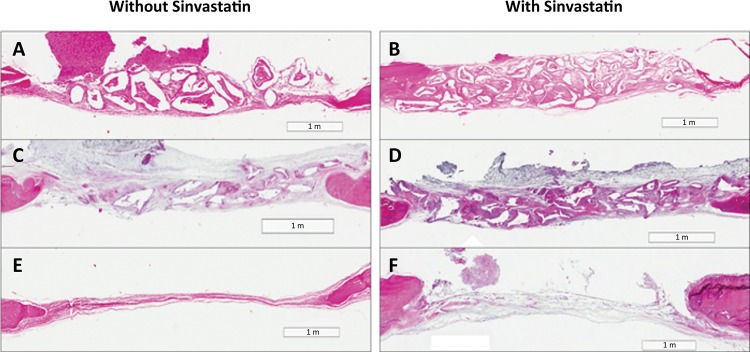
Representative images of the entire defects (HE-x25). A and B represent DBB; C and D represent HA/TCP; E and F represent CS. All groups showed that the bone formation begins at the edges of the critical-sized calvaria defects to the center. However, there was no complete closure of the defects observed in this study, which confirms that the defect made in this study was critical. In addition, the CS group presented fewer on low remnants of the sponge, which is responsible for the poor volume of repaired tissue found in the defect area compared with the groups of the DBB and HA/TCP. The addition of SVT improved the bone formation in all the groups as determined by the histometric analysis.

In the histometric analysis ( Fig. 2 ), the use of SVT showed to increase the bone formation in CSDs when combined with all the bone substitutes tested (p<0.05). In addition, the use of SVT reduced resorption of HA/TCP particles (p<0.05). Among the biomaterials used, DBB induced the greatest formation of bone tissue, followed by HA/TCP and CS when used alone or when combined with SVT. DBB was also the biomaterial that presented the greatest percentage of particle remnants (p <0.05), while CS was totally degraded (p <0.05).

## Discussion

In general, this study confirmed previous findings that SVT has the potential to induce bone tissue formation because all groups in which SVT was combined with different biomaterials used to fill CSDs (DBB, HA/TCP and CS) showed greater bone tissue formation than CSDs that were grafted with the same biomaterials alone.

These results corroborate a range of studies that have demonstrated beneficial effects of SVT on bone tissue metabolism ^17 , 24 , 27^ . Studies on periodontal disease in rats demonstrated that SVT has both a protective effect against the destructive inflammatory process ^17 , 28^ and is also capable of inducing the formation of bone tissue ^29 , 30^ . In fact, SVT has the potential to block osteoclastogenesis ^19^ . This effect is related to its action as a blocker of the mavelonate pathway, which, in addition to reducing cholesterol conversion, also reduces the formation of osteoclasts ^16 , 28^ . In addition, SVT has shown to reduce the expression of proinflammatory cytokines, such as IL-6, IL-8, TNFα and IL-1β ^16 , 17^ . Conversely, SVT-induced bone formation found in this study may be related to its effect on increasing the expression of BMP2, VEGF and alkaline phosphatase, which are biological markers of osteoblastic differentiation and function ^17 , 20 , 30^ .

However, the real potential of using SVT clinically as an inducer of bone tissue formation is still highly questionable because not all studies have shown that this substance has a protective effect on bone tissue ^23^ . Some factors may influence these conflicting data regarding the use of SVT; one such factor is the route of administration. Studies in humans have shown that the systemic use of SVT mildly increases bone density in patients with osteoporosis. As high systemic doses of SVT have been related to myopathic and hepatic complications, the systemic use of this drug to improve bone tissue metabolism is not recommended ^28^ . For this reason, we decided to carry out this study using a local application of SVT combined with biomaterials.

In this study, the defects in which the biomaterials were used in combination with SVT had a greater degree of inflammation than did the groups in which the biomaterials were used alone. These findings corroborate findings from other studies showing that the use of SVT locally increases inflammatory infiltrate ^23 , 24^ . However, at the dose used, despite the increase in the inflammatory infiltrate, bone tissue formation still increased ^24^ , as observed in this study. SVT doses greater than 0.25 mg applied locally have shown to prevent bone formation, whereas this effect was also observed for doses less than 0.1 mg ^24^ . Thus, the dose used in this study is within the SVT dose range that induces bone tissue formation without the production of exacerbated inflammatory infiltrate ^24^ .

Another aspect to be discussed is the type of vehicle that may be associated with the use of SVT. The use of a vehicle allows for the controlled release of SVT in the medium for a greater amount of time ^22^ . In fact, methylcellulose gel polymers and gelatin/nanohydroxyapatite allow the release of SVT in the medium for more than 15 days, which is the time that is essential for bone tissue formation ^20 , 27^ . In the present study, no vehicle was used to control the release of SVT, and no bone formation inhibition was observed in the groups applying the biomaterials combined with the drug. The use of a vehicle does not reduce the costs of the materials; however, it is possible that if SVT was attached to the biomaterials tested using a vehicle, the effects could have been even more positive.

In this study, DBB promoted the greatest bone tissue formation when combined with SVT or when used alone, followed by the HA/TCP biphasic ceramics. In addition, DBB was the biomaterial with the greatest amount of particle remnants, which shows that the maintenance of the space required for newly formed bone promoted by the presence of these particles on the defect may have been an important factor for bone tissue formation ^6 , 18^ . A pre-clinical study evaluating the effect of SVT combined with DBB, calcium sulphate and CS on bone repair in rabbit tibial defects showed that DBB was the biomaterial that promoted the greatest bone tissue formation after 4 weeks ^18^ . Thus, as observed in the present study, the work of Papadimitriou *et al* . ^18^ , also demonstrated that the use of CS combined with SVT was the group the worst results regarding bone tissue formation. This was confirmed in a study that evaluated the effect of SVT combined with CS on the repair of critical size defects in rat calvaria; this study showed that this combination did not benefit bone tissue formation. It is likely that rapid rates of CS resorption will not allow the maintenance of the space required for the newly formed bone tissue ^18^ .

The results of this study must be interpreted with caution because of the study’s limitations. Although the use of 0.1 mg SVT benefited bone formation, it was not attached to the biomaterials using a vehicle, and it is possible that a more controlled release of SVT could benefit the formation of bone tissue over a longer period. The biomaterials used were either of slow resorption (DBB and HA/TCP) or very fast resorption (CS), and SVT has been shown to promote good results when combined with polymeric scaffolds that are likely to degrade at a more favourable rate that would improve the quality of the newly formed bone tissue ^20^ . However, the use of SVT without the need for a vehicle and combined with a commercially available biomaterial may be a cheaper way to potentiate the formation of bone tissue without the need to produce new biomaterials.

## Conclusions

The use of SVT, therefore, stimulated the formation of bone tissue regardless of the type of biomaterial it was combined with (DBB, HA/TCP and CS). Among the biomaterials evaluated, DBB resulted in the greatest potential for bone tissue formation despite being less resorbable than the other biomaterials.
